# Chinese Medicine Injection Shuanghuanglian for Treatment of Acute Upper Respiratory Tract Infection: A Systematic Review of Randomized Controlled Trials

**DOI:** 10.1155/2013/987326

**Published:** 2013-03-30

**Authors:** Hongwei Zhang, Qin Chen, Weiwei Zhou, Shi Gao, Huiguang Lin, Shuifen Ye, Yihui Xu, Jing Cai

**Affiliations:** ^1^School of Chinese Medicine, Faculty of Science, The Chinese University of Hong Kong, Shatin, New Territories, Hong Kong; ^2^The Affiliated People's Hospital of Fujian University of Traditional Chinese Medicine, Fuzhou, Fujian 350004, China; ^3^College of Traditional Chinese Medicine, Fujian University of Traditional Chinese Medicine, Fuzhou, Fujian 350122, China; ^4^Academy of Integrative Medicine, Fujian University of Traditional Chinese Medicine, Fuzhou, Fujian 350122, China

## Abstract

Acute upper respiratory tract infections (AURTIs) are the illnesses caused by an acute infection with various viruses and bacteria involving the upper respiratory tract. Shuanghuanglian (SHL) injection, a Chinese medicine intravenous preparation extracted from honeysuckle, *Scutellaria baicalensis*, and fructus forsythiae, is commonly used to treat AURTIs. Although it is used largely in Chinese hospitals, there is no substantial evidence to demonstrate its clinical effect on AURTIs. We conducted a systematic review to evaluate the effectiveness and safety of Shuanghuanglian injection for the treatment of acute upper respiratory tract infections.

## 1. Introduction

Acute upper respiratory tract infections (AURTIs) are the illnesses caused by an acute infection with various viruses and bacteria involving the upper respiratory tract. They include the common cold, laryngitis, pharyngitis/tonsillitis, acute rhinitis, acute rhinosinusitis, and acute otitis media, which are the commonest acute problem dealt with in primary care [1, 2]. Symptoms of AURTIs commonly include cough, sore throat, runny nose, nasal congestion, headache, low grade fever, facial pressure, and sneezing. Although the available evidence has shown that antibiotics probably provide little benefit for a large proportion of respiratory tract infections, antibiotics are still largely inappropriately used in clinic [3–5]. Antibiotic treatment to prevent suppurative and nonsuppurative complications may be inappropriate nowadays with generally low rates of major complications [3]. More effective approaches to control infections and relieve symptoms in AURTIs are in a great need.

Shuanghuanglian (SHL) injection, a Chinese medicine intravenous preparation extracted from honeysuckle, *Scutellaria baicalensis*, and fructus forsythiae [6], is commonly used to treat various kinds of infectius diseases caused by bacterium or viruses in respiratory traction [7]. The main chemical components of SHL injection are chlorogenic acid, baicalin, and forsythia glycosides [8], which have been found to have the ability of anti-inflammation, improving immunity, and inhibiting the growth of various viruses [9–11]. It has been reported that SHL injection could inhibit the respiratory syncytial viruse, parainfluenza I–IV [12], and 23 kinds of pathogenic bacteria such as *Staphylococcus aureus* [13] and *Pseudomonas aeruginosa* [14]. SHLI can also enhance the NK cell activity, promote the production of alpha-interferon, raise the rate of lymphocyte transformation [15], and decrease the level of CD4+ cells and the ratio of CD4+/CD8+ while increasing CD8+ [16]. 

SHL injection has been approved for treatment of acute respiratory tract infection since 1973 in China [17]. Although it is used largely in Chinese hospitals, there is no substantial evidence to demonstrate its clinical effect on AURTIs. We conducted a systematic review to evaluate the effectiveness and safety of Shuanghuanglian injection for the treatment of acute upper respiratory tract infection. 

## 2. Methods

### 2.1. Inclusion Criteria

We included randomized controlled trials evaluating SHL injection for the treatment of AURTIs without language or publication status restriction. Any patients with AURTIs, including common cold, laryngitis, pharyngitis/tonsillitis, acute rhinitis, acute rhinosinusitis, and acute otitis media, without limitation on gender and age were included in the review. We defined the interventions as Shuanghuanglian injection in the form of liquid or power in the intravenous route of administration. The control group may have a placebo, nontreatment, or conventional treatment. Cointerventions such as supportive or symptomatic treatment were allowed as long as all arms of the randomized trial received the same cointervention(s). We excluded studies on other administration routes of Shuanghuanglian, comparing SHL injection with other Chinese herbal medicine, or SHL injection combined with other antibiotics or antivirus medication.

For trials to be eligible for this review, their results need to be extracted on at least one of the following primary outcomes: (1) severity of symptoms; (2) time to resolution of some common acute URTI-related symptoms (e.g., fever, cough, nasal discharge, cough, congestion, sneezing, and headache) and (3) one of the secondary outcomes: resolution of fever in five days, time off from school or work, antibiotic use, and adverse events associated with treatment.

### 2.2. Databases and Search Strategies

We searched the following electronic databases: Medline (1950 to 2012), Embase (1980 to 2012), the Cochrane Central Register of Controlled Trials (Issue 10, 2012), AMED (Allied and Complementary Medicine Database; 1985 to 2012), CMCC (Chinese Medical Current Contents, 1994 to 2012), China National Knowledge Infrastructure (CNKI) (1979 to 2012), VIP Database for Chinese Technical Periodicals (VIP) (1989 to 2012), and Wanfang Med Database (1994 to 2012). We employed highly sensitive strategies in which adapted subject headings and text words were developed around Shuanghuanglian and upper respiratory infection. Within these text words they were combined with “or,” and then the two kinds of searching terms were combined with “and.” For Chinese databases searching, additional limit on the study type of randomized controlled trial was added. Reference lists of included studies and significant reviews were also checked.

### 2.3. Data Extraction and Quality Assessment

Two authors (W. Zhou and S. Gao) independently screened the titles and abstracts of the search results to identify potential relevant studies. If necessary, their full texts were obtained for further evaluation on inclusion criteria. These two authors independently used self-developed data extraction form to extract data regarding study methods, participants, interventions, outcomes, and results. Any discrepancies were resolved by discussion between the two reviewers. 

To assess the study quality, we used risk of bias assessment tool recommended by the Cochrane Collaboration to address the following six domains: sequence generation, allocation concealment, blinding, incomplete outcome data, selective outcome reporting, and “other issues” [18]. The baseline comparability was considered in the “other issues.” The risk of bias for each outcome within and across the included studies was summarized into three levels: low, unclear, and high risk of bias. We used GRADE system to further assess the quality of the evidence for each individual outcome across included studies. Besides within-study risk of bias (methodological quality), the GRADE approach incorporates considerations of directness of evidence, inconsistency or heterogeneity, precision of effect estimates, and risk of publication bias [18, 19]. 

### 2.4. Data Analysis and Synthesis

We used risk ratio (RR) with 95% confidence intervals (CI) to summarize dichotomous outcome data of individual studies and used Mantel-Haenszel random-effects model to pool the results across all included studies. We used the mean difference (MD) to summarize continuous outcome data at the end of treatment or followup within studies and used the inverse-variance random-effects model to pool the results across studies. For meta-analysis, we used random-effects model because of the expected heterogeneity of the interventions. We examined forest plot visually first to detect heterogeneity and then used chi-squared test with an alpha of 0.1 for statistical significance and *I* ^2^ test to analyze heterogeneity across the included studies. We conducted funnel plot to investigate the possibility of publication bias. 

## 3. Results

### 3.1. Search Results and Trial Characteristics

**Figure 1:**
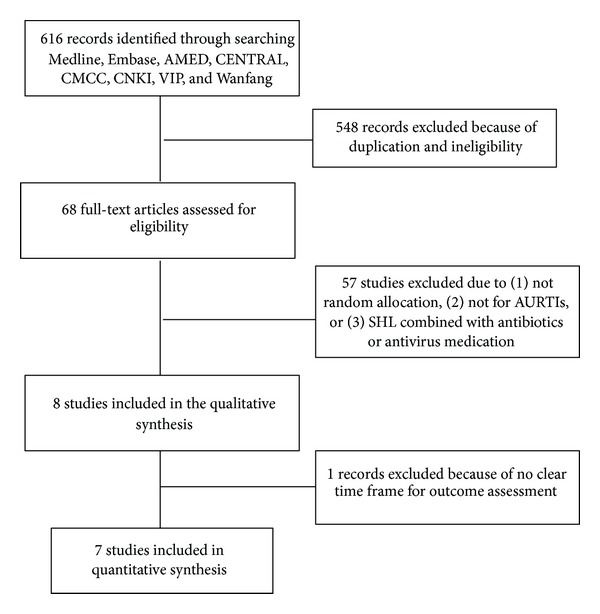
Flow chart showing the search process and study selection.

The flow chart in Figure 1 depicts the search process and study selection. We screened the title or abstract of 616 studies and assessed the full texts of 68 papers in Chinese or English. A total of 8 trials were finally included in the systematic review involving 857 participants, of whom 497 (58%) were males and 360 (42%) were females [20–27]. All these trials were conducted in China and published unanimously in Chinese.

Table 1 summarizes the baseline characteristics of the patients. Of the 8 trials, 3 compared SHL injection with penicillin [22, 23, 25], another 3 compared SHL injection with ribavirin [20, 21, 26], and the other 2 studies compared SHL injection with penicillin and ribavirin [24, 27]. The treatment duration was generally 3 or five days except one. It was stated that treatment was provided for 3 to 7 days [27], so it was not included into the meta-analysis.

### 3.2. Trial Quality

We used the Cochrane Collaboration's tool for assessing risk of bias and GRADE system to assess the quality of evidence pertaining to each individual outcome. The randomized allocation was stated all the included studies. After contacting trial authors by telephone, it was confirmed that 4 studies used random number table or computer to generate random numbers [21–23, 25]. In other 4 studies, the authors could not be accessed to obtain further information about the method for randomization [20, 24, 26, 27]. Allocation concealment was not mentioned in all studies.

No measures for blinding were ever mentioned in the included studies. The outcome measures were collected and recorded immediately after 3 or five days of treatment. No missing data was found in all the included studies. Selective reporting was generally unclear in the included studies because no information about the protocol was available. The baseline characteristics were in general comparable within each of the studies except the imbalance in the numbers between treatment and control group in two studies [24, 27]. 

Based on the summarization of six domains on methodological evaluation, the risk of bias within studies was unclear in 4 studies [21–23, 25] and high in the other 4 studies [20, 24, 26, 27]. 

### 3.3. The Effect of the Interventions

#### 3.3.1. Primary Outcomes

**Figure 2:**
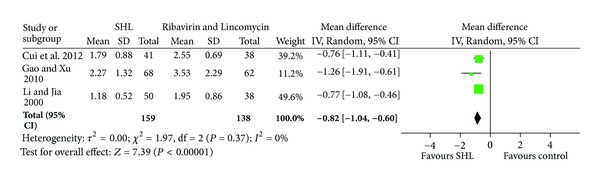
Effects of SHL injection on the time to resolution of fever when compared with Ribavirin and/or Lincomycin.

**Figure 3:**
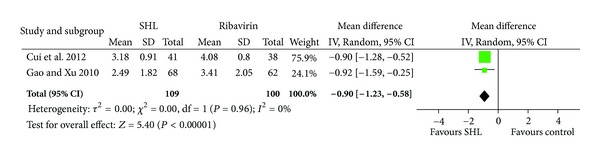
Effects of SHL injection on the resolution time of cough when compared with Ribavirin.

*Time to Resolution of Some Common Acute URTI-Related Symptoms. *SHL injection showed significant effect on reducing the time to resolution of fever (3 trials, 297 patients; MD 0.82 day, 95% CI 0.6 to 1.04) and the resolution time of cough (2 trials, 209 patients; MD 0.9 day, 95% CI 0.58 to 1.23), when compared with ribavirin and/or lincomycin (Figures 2 and 3).

It was also reported that SHL injections had significant effect on reducing the resolution time of sore throat (1 trial, 79 patients; mean difference 1.39 day, 95% CI 0.88 to 1.9) and nasal congestion and discharge (1 trial, 130 patients; mean difference 0.74 day, 95% CI 0.11 to 1.37).

#### 3.3.2. Secondary Outcomes

**Figure 4:**
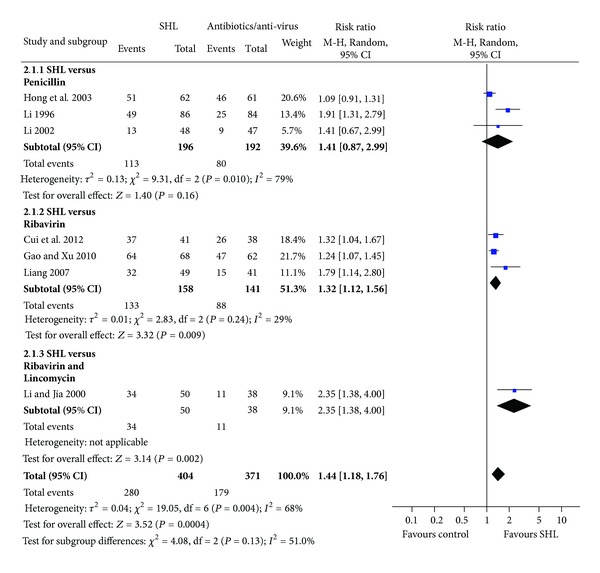
Effects of SHL injection on the fever resolution when compared with antibiotics/anti-virus.

*Fever Resolution in Five Days.* We also found a significant effect of SHL injection on reducing the incidence of fever resolution in five days (7 trials, 775 patients; relative risk 1.44, 95% CI 1.18 to 1.76), when compared with ribavirin and/or penicillin. A moderate heterogeneity was found in this analysis (*I* ^2^ = 68%) (Figure 4). Different treatment duration, control interventions, and disease severity of patients may contribute substantially to this heterogeneity. Subgroup analysis on different control interventions generated similar results. 

Although publication bias was not detected by funnel plot analysis because of no sufficient number of included studies, it should be noticed during the interpretation of meta-analysis results.

*Adverse Effects.* Adverse effects were reported in 5 included studies and were not described in the other 3 studies [22, 23, 25]. Abdominal distension, diarrhea, nausea, and vomiting was reported in 4 studies in the treatment group and relieved after symptomatic treatment. Skin rash was found in 6 among 50 patients in the treatment group after receiving the first SHL injection treatment and soon relieved after antihistamine treatment [24].

## 4. Discussion

We found in this systematic review that Shuanghuanglian injection showed better effect than common antibiotics on helping relieve some symptoms, such as fever, cough, sore throat, and nasal congestion and discharge and decrease the course of acute upper respiratory tract infections. However, due to the limited number of reports on primary outcomes and generally low methodological quality of included studies, no definite conclusion can be drawn on the effect of SHL injection on AURTIs. 

According to Chinese medicine, Shuanghuanglian has the effect of clearing away heat and toxic material and is suitable for diseases caused by heat and toxins. Most AURTIs are differentiated in Chinese medicine as syndrome of heat; however, some complications such as insufficiency in Qi, Yin, or Yang may exist. In this paper, there was no description of syndrome of AURTIs patients in all the included studies. Therefore, it is difficult for us to further explore the effect of SHL for different syndromes of AURTIs. As a common medicine for AURTIs, Shuanghuanglian was prepared in different forms including oral tablet or granule; the injection administered intravenously provides us an approach to deliver the medicine more quickly. However, safety issues about SHL injection should be paid much attention. Some adverse effects related to SHL injection may be due to impurities from the production procedure. The difference in oral SHL or SHL injection may be examined further to explore a more safe, effective, and efficient treatment approach.

For the outcome reporting, most of the studies published in China used the national evaluation criteria for Chinese medicine research. A composite outcome measure, effective rate or noneffective rate, which combines the temperature, several clinical symptoms or signs such as pharyngeal check-up, and blood counting, was reported in some of the included studies. It is somewhat difficult for interpretation. Therefore, the outcome of fever resolution in five days was chosen from the composite measurement. However, it should be noted that in some studies this outcome was reported in three days and not measured separately. It may be somewhat different from the detection of single symptom. In addition, the measure of time to resolution of some common acute URTI-related symptoms, such as fever, cough, sore throat, and nasal congestion and discharge, was reported in some studies. However, no detailed information on how to measure them was reported. The subjective influence, especially when no blinding measurement was taken, cannot be ignored when interpreting these results.

In some included studies, cointerventions such as supportive or symptomatic treatments were provided in both treatment and control groups. Some analgesics and antipyretics drugs, cough suppressants, were administered as cointerventions in the two groups. In the situation of unblinding during the trial, it could be excluded that the cointerventions may be different between groups.

In conclusion, Shuanghuanglian injection may have potential effect on relieving some symptoms such as fever, cough, and sore throat and reduce the disease course in acute upper respiratory tract infections. Due to the methodology weakness of the included studies and often poor reporting, this paper cannot make any definite conclusions regarding the clinical effectiveness of SHL injection for AURTIs. 

The available clinical evidence and related pharmacological findings so far highlight the need for further research on SHL injection for AURTIs. In particular, the randomized controlled trials with scientifically rigorous methodology are badly needed. To make a more comprehensive understanding on the potential effect of SHL injection, the outcomes, such as severity of symptoms, time to resolution of some common acute URTI-related symptoms, or antibiotic use, should be measured. Furthermore, possible adverse effect associated with the use of SHL injection should also be an important issue of investigation. 

## Figures and Tables

**Table 1:** Characteristics of included studies.

Study ID	Number (male/female)	Treatment/control	Disease	SHL intravenous injection, dosage	Treatment duration	Control	Outcome measure
Li 1996 [23]	103/67	86/84	Acute tonsillitis	60 mg/kg/d (injection powder)	3 d	Penicillin	Fever resolution
Li and Jia 2000 [24]	35/53	50/38	Acute upper respiratory infection	1 mL/kg/d (injection)	3 d	Ribavirin and lincomycin	Fever resolution after treatment; time to resolution of fever
Li 2002 [25]	53/42	48/47	Acute tonsillitis	60 mg/kg/d (injection powder)	5 d	Penicillin	Fever resolution
Hong et al. 2003 [22]	73/50	62/61	Acute tonsillitis	60 mg/kg/d	5 d	Penicillin	Fever resolution
Zhang and Hu 2003 [27]	45/37	52/30	Acute upper respiratory infection	1 mL/kg/d, 20 mL (maximum) (injection)	3–7 d	Penicillin and ribavirin	No clear description
Liang 2007 [26]	54/36	49/41	Acute upper respiratory infection	60 mg/kg/d (injection powder)	5 d	Ribavirin	Fever resolution
Gao and Xu 2010 [21]	77/53	68/62	Acute upper respiratory infection	60 mg/kg/d (injection powder)	5 d	Ribavirin	Fever resolution after treatment; time to resolution of fever, cough, and nasal congestion and discharge
Cui et al. 2012 [20]	57/22	41/38	Acute upper respiratory infection	60 mg/kg/d (injection powder)	5 d	Ribavirin	Fever resolution after treatment; time to resolution of fever, cough, and sore throat
